# Correction: Application of [PVI-SO_3_H]NO_3_ as a novel polymeric nitrating agent with ionic tags in preparation of high-energetic materials

**DOI:** 10.1039/d1ra90097h

**Published:** 2021-04-06

**Authors:** Hassan Sepehrmansourie, Mahmoud Zarei, Mohammad Ali Zolfigol, Amin Mehrzad, Hamid Reza Hafizi-Atabak

**Affiliations:** Department of Organic Chemistry, Faculty of Chemistry, Bu-Ali Sina University Hamedan 6517838683 Iran mahmoud8103@yahoo.com zolfi@basu.ac.ir mzolfigol@yahoo.com +988138380709 +988138282807; Chemistry and Chemical Engineering Research Center of Iran Tehran 14335-186 Iran

## Abstract

Correction for ‘Application of [PVI-SO_3_H]NO_3_ as a novel polymeric nitrating agent with ionic tags in preparation of high-energetic materials’ by Hassan Sepehrmansourie *et al.*, *RSC Adv.*, 2021, **11**, 8367–8374, DOI: 10.1039/D1RA00651G.

The authors regret that incorrect details were given for ref. 16 in the original article. The correct version of ref. 16 is given below as ref. 1.

The Royal Society of Chemistry apologises for these errors and any consequent inconvenience to authors and readers.

## Supplementary Material
